# Association of serum irisin and body composition with chronic kidney disease in obese Chinese adults: a cross-sectional study

**DOI:** 10.1186/s12882-015-0009-5

**Published:** 2015-02-11

**Authors:** Shuyu Yang, Fangsen Xiao, Lingling Pan, Huijie Zhang, Zhimin Ma, Suhuan Liu, Yongwen Liu, Wei Zhang, Xin Zeng, Changqin Liu, Xiaoying Li, Xuejun Li, Zhibin Li

**Affiliations:** Xiamen Diabetes Institute, Xiamen, China; Department of Endocrinology and Diabetes, The First Affiliated Hospital, Xiamen University, 55# Zhenhai Road, Xaimen, 361003 China; Shanghai Institute of Endocrinology and Metabolism, Rui-Jin Hospital, School of Medicine, Shanghai Jiaotong University, Shanghai, China; Department of Endocrinology, The Second Affiliated Hospital of Soochow University, Suzhou, China; Department of Nursing, The First Affiliated Hospital, Xiamen University, Xiamen, China; Epidemiology Research Unit, The First Affiliated Hospital, Xiamen University, No.55 Zhenhai Road, Xaimen, 361003 China

**Keywords:** Irisin, Body composition, Chronic kidney disease, Epidemiology, Cross-sectional study

## Abstract

**Background:**

Irisin, an exercise induced myokine, has broad implications for metabolism and energy homeostasis. Available evidence about the association of serum irisin with chronic kidney disease (CKD) is limited.

**Methods:**

Cross-sectional data on socio-demographic, lifestyle, clinical characteristics and serum irisin were collected for 1,115 community-living obese Chinese adults (waist circumference ≥ 90 cm for men and ≥ 80 cm for women). CKD was defined as estimated glomerular filtration rate less than 60 ml/min per 1.73 m^2^ and/or the presence of albuminuria. Associations of serum irisin and body composition measurements with CKD were analyzed using multivariable logistic regression.

**Results:**

The overall prevalence of CKD were 23.1% (26.6% in females and 15.5% in males, p < 0.001). Subjects within quartile 4 group of serum irisin had significantly the lowest prevalence of CKD (22.9%, 22.2%, 28.7% and 18.7% for quartile 1–4 groups, respectively, p = 0.046). With adjustment for potential confounders, compared with those within quartile 1 group of serum irisin, subjects within quartile 4 group showed significantly decreased risk of CKD and marginally decreased risk of albuminuria, with the adjusted odds ratios (ORs, 95% CI) of 0.572 (0.353-0.927, p = 0.023) and 0.611 (0.373-1.000, p = 0.050), respectively. As for body composition measurements, only body fat percentage was significantly associated with both albuminuira and CKD, with ORs (95% CI) of 1.046 (1.002-1.092, p = 0.039) and 1.049 (1.006-1.093, p = 0.025), respectively. No statistically significant interaction effect between serum irisin and body composition measurements on CKD was found.

**Conclusions:**

Our results imply that high serum irisin level was associated with reduced risk of CKD, and should be confirmed in future studies. Furthermore, adiposity per se, rather than body weight or body shape, is independently associated with increased risk of CKD. Future studies should examine whether decreasing body fat percentage may prevent or slow CKD.

## Background

Chronic kidney disease (CKD), typically defined by reduced estimated glomerular filtration rate (eGFR) and/or albuminuria, is considered as a major public health problem because of its increasing prevalence worldwide and its independent association with all-cause mortality and cardiovascular mortality and progression to end stage renal disease [1-4]. The prevalence rate of CKD in China is about 10% [5-7]. Understanding the risk factors of CKD and identifying individuals with specific risk factors remain important prevention strategies.

Obesity has become the largest epidemic of modern time and is associated with an increased risk of insulin resistance, metabolic syndrome, type 2 diabetes, hypertension, cardiovascular disease, cancer and CKD [8,9]. Exercise has long been consistently shown to be effective in improving body composition and metabolic health [10,11], although the underlying mechanisms for the clinical benefits of exercise remain to be fully elucidated. Boström recently found that irisin, a putative exercise induced myokine, drives brown-fat-like conversion of white adipose tissues, and has broad implications for metabolism and energy homeostasis [12,13]. Irisin is secreted by myocytes and proteolytically processed from the product of the FNDC5 gene prior to being released into the circulation and regulated by PPAR-γ coactivator-1(PGC1)-α. In mice, FNDC5 expression in muscle and circulating irisin increased in response to overexpression of PGC1-α as well as 3 weeks of aerobic exercise-training [12]. Huh also found circulating irisin levels were significantly upregulated 30 min after acute exercise [14]. Because low serum irisin was found to be associated with type 2 diabetes and obesity [15-17] and both these two conditions were independent risk factors of CKD [1], it is suspected that CKD patients have altered irisin levels. However, evidence on the association between serum irisin and CKD is limited with complicated results [18-20], and more studies are awaited to validate this association.

Most previous studies have found that obesity is independently associated with increased risk of CKD, and body mass index (BMI) has been widely used as the index of obesity in these studies [1-3,5-7]. However, numerous studies have reported that BMI and other measures of adiposity, such as waist circumference, body fat percentage, have been shown to have different associations with various health conditions [21-23]. Recent studies show that irisin is not only a myokine but also an adipokine [24,25]. Therefore, more studies are warranted to test the independent association of serum irisin concentration and body composition measurements with CKD in different populations.

In the present cross-sectional study of 1,115 community-living healthy obese Chinese adults, we aimed to determine the independent association of serum irisin concentration and different body composition measurements with CKD. Furthermore, potential interaction effects between serum irisin and body composition measurements with CKD were tested.

## Methods

### Study population

Obese adults were local residents aged 40 years or older living in the Lianqian community, Xiamen, China, and were screened with physical examination from April 2011 to August 2011. Subject sampling, recruitment and evaluation have been described earlier [26,27]. Briefly, a total of 1,523 subjects with central obesity (waist circumference greater than 90 cm for men and 80 cm for women) were included. Of them, 1,115 (73.2%) subjects with the complete data on the entire examination were left for further analysis. The study was approved by the Human Research Ethics Committee of the First Affiliated Hospital of Xiamen University (Xiamen, China). Written informed consent was obtained from each participant.

### Measurements

Screening protocol and evaluation criteria were described elsewhere [26,27]. Staff members participating in this study are doctors and medical students, who received intensive training for epidemiologic screening methods. Data were collected at community health service centers. Standard questionnaires were used during face-to-face interview to collect socio-demographic status, lifestyle habits (including physical activities using the International Physical Activity Questionnaire –Long form), present and previous health history and medications. Subjects, who had cancer, current treatment with systemic corticosteroids, biliary obstructive diseases, acute or chronic virus hepatitis, drug-induced liver diseases, total parenteral nutrition, autoimmune hepatitis, Wilson’s disease, known hyperthyroidism or hypothyroidism, were excluded.

#### Anthropometric and clinical measurements

Anthropometric measurements were obtained using standard protocols and techniques. After removal of shoes and heavy clothing, each subject underwent weight, height and waist circumference measurements, using a calibrated scale. Body mass index (BMI) was calculated as weight in kilograms divided by height in squared meters as a measure of general obesity. Waist circumference was measured at the midpoint between the inferior costal margin and the superior border of the iliac crest on the midaxillary line. Body fat were quantified with the Hologic whole body DXA systems (Hologic Inc., Bedford, MA). Arterial blood pressure was measured with a mercury sphygmomanometer after sitting for at least 15 minutes. Blood pressure measurements were taken according to the Joint National Committee VII criteria (JNC VII) [28]. Three readings were taken at 5-min intervals. The mean of the three measurements was recorded.

#### Biochemical measurements

75-g oral glucose tolerance test and blood biochemical measurements were conducted for each subject. All blood samples were obtained after 12-hour fasting. Blood and urine samples were refrigerated at −20°C, transferred and tested in the central laboratory of the First Affiliated Hospital, Xiamen University. Plasma glucose, liver enzyme levels, and serum lipid profiles, including triglyceride (TG), total cholesterol (TC), and high-density lipoprotein cholesterol (HDL-C) were determined on a HITACHI 7450 analyzer (HITACHI, Tokyo, Japan). Low-density lipoprotein cholesterol (LDL-C) was calculated by Friedewald’s formula. Fasting plasma glucose concentration (FPG) and 2-hour plasma glucose concentration (2hPG) were measured by the hexokinase method. Serum fasting insulin concentration was measured by electrochemiluminiscence immunoassay (Roche Elecsys Insulin Test, Roche Diagnostics, Mannheim, Germany). HOMA-insulin resistance (HOMA-IR) was calculated by fasting serum insulin (Flns, mU/ml) *fasting blood glucose (FPG, mmol/L)/22.5. Raised fasting plasma glucose (FPG) was defined as FPG ≥ 100 mg/dL (5.6 mmol/L) or previously diagnosed type 2 diabetes. Serum uric acid was measured by the auto-analyzer (COBAS INTEGRA 400 plus, Roche, Basel, Switzerland); and hyperuricemia was defined as the serum uric acid level >7.0 mg/dL in males and >6.0 mg/dL in females.

#### Serum irisin measurement

Serum irisin concentration was measured using the enzyme-linked immunosorbent assay (ELISA) kits (Aviscera Biosciences, Santa Clara, CA). The assay was proven to be highly sensitive to human irisin [26,27]. The sensitivity of the assay was 0.2 ng/ml and the linear range of the standard was 5 to 500 ng/ml. The intra- and inter- assay variations were both less than 10%.

#### Definition of chronic kidney disease

Serum creatinine (Scr) was measured by Jaffe’s kinetic method and was standardized at the central laboratory to a national reference. Estimated glomerular filtration rate (eGFR) was calculated using the following estimating equation which was developed by modifying the Modification of Diet in Renal Disease (MDRD) equation based on the data from Chinese CKD patients [29,30].

$$ eGFR\ \left( mL/ min/1.73\ {m}^2\right) = 175 \times Scr{\left( mg/ dL\right)}^{-1.234} \times age{(year)^{-}}^{0.179}\left[ female \times 0.79\right] $$

Reduced renal function was defined as an eGFR <60 mL/min/1.73 m^2^.

Urinary creatinine and albumin were measured on a morning urine sample using an automatic analyzer (COBAS INTEGRA 400 plus, Roche, Basel, Switzerland). Creatinine was measured using the same method as that for serum creatinine. Urinary albumin was measured by immunoturbidimetric method. Urinary albumin-to-creatinine ratio (ACR, milligram per gram) was calculated. Microalbuminuria and macroalbuminuria were defined according to the guideline of American Diabetes Association as an increase in ACR between 30 and 299 mg/g and 300 mg/g or over, respectively. The term of albuminuria was used to describe the presence of either microalbuminuria or macroalbuminuria. CKD was defined as the presence of either a reduced glomerular filtration rate or albuminuria [1].

### Statistical analysis

Data were presented as the mean ± standard deviation or median (interquartile range) for continuous variables or number and percentage for categorical variables. Irisin was also log-transformed to obtain better approximation of normal distribution. Differences between subjects were analyzed using one way ANOVA or Mann–Whitney U test for continuous variables and chi-square test for categorical variables.

Multivariable logistic regression was used to calculate adjusted odds ratios (OR) and 95% confidence intervals (CI) of serum irisin concentration and body composition measurements for reduced renal function, albuminuria and CKD in different models with adjustment for potential confounders. In model 1, age and sex were adjusted for; in model 2, educational level, smoking and drinking habits and physical activity were further adjusted for; in model 3, SBP, DBP, serum globulin, hyperuricemia, TG, LDL and FPG. Different measurements of body composition, such as BMI, waist circumference, body fat mass and body fat percentage, were tested in the multivariable logistic regression models separately. In each of the multivariable logistic regression models, interaction effects among serum irisin, body composition measurements and diabetes status were tested separately; and stratified analysis by diabetes status was conducted as well. P <0.05 was considered statistically significant. All statistical analyses were performed using R version 3.1.0 [31].

## Results

Of the 1,115 participants, 766 (68.7%) were female. The mean age (±SD) of women and men were 53.1 (±7.1) years and 53.3 (±7.6) years (p = 0.702), respectively. Females had higher prevalence of CKD (26.6% vs. 15.5%, p < 0.001) and albuminuria (23.8% vs. 12.6%, p < 0.001), but showed similar prevalence of reduced renal function (3.8% vs. 3.2%, p = 0.598), when compared with males. The total prevalence rates (95% confidence interval (CI)) of CKD were 26.6% (23.2%-29.4%) in females and 15.5% (11.8%-19.2%, p < 0.001) in males, respectively.

### Demographic and clinical characteristics stratified by reduced renal function, albuminuria and CKD

Differences in demographics, life style habits, body composition measurements and clinical characteristics of subjects stratified by reduced renal function, albuminuria and CKD are presented in Table 1. Older age was significantly associated with higher prevalence of reduced renal function, albuminuria and CKD. Generally, subjects with albuminuria and CKD showed significantly higher levels of systolic BP, diastolic BP, serum globulin, TG, total cholesterol, LDL, fasting glucose, fasting insulin, HOMA-IR and HbA1c than their controls. Patients with reduced renal function showed significantly higher systolic BP, serum globulin and blood uric acid than their controls. As for body composition measurements, subjects with albuminuria and CKD showed significantly higher levels of BMI, waist circumference, body fat mass and body fat percentage but lower levels of muscle mass and body fat free mass than their controls. There was no significant difference in serum irisin levels (neither the original serum irisin values or the corresponding log-transformed values) for subjects with reduced renal function, albuminuira and total CKD compared with their controls.

**Table 1:** **Demographic, lifestyle and clinical characteristics of subjects by reduced renal function, albuminuira and CKD**

	**Reduced renal function**			**Albuminuira**			**CKD**		
**Variables**	**No**	**Yes**	**P value**	**No**	**Yes**	**P value**	**No**	**Yes**	**P value**
**Demographics**									
N (%)	1075 (96.4%)	40 (3.6%)		889 (79.7%)	226 (20.3%)		857 (76.9%)	258 (23.1%)	
Sex			0.6			<0.001‡			<0.001‡
Female (n, %)	737 (68.6%)	29 (72.5%)		584 (65.7%)	182 (80.5%)		562 (65.6%)	204 (79.1%)	
Male (n, %)	338 (31.4%)	11 (27.5%)		305 (34.3%)	44 (19.5%)		295 (34.4%)	54 (20.9%)	
Age (years)	53.0 ± 7.1	58.9 ± 6.7	<0.001‡	52.8 ± 7.2	54.6 ± 7.2	0.002†	52.6 ± 7.1	55.1 ± 7.3	<0.001‡
Education categories, (n, %)			0.2			0.1			0.4
Illiteracy	292 (27.2%)	10 (25.0%)		227 (25.5%)	75 (33.2%)		221 (25.8%)	81 (31.4%)	
Elementary school	324 (30.1%)	6 (15.0%)		263 (29.6%)	67 (29.7%)		257 (30.0%)	73 (28.3%)	
Middle school	243 (22.6%)	13 (32.5%)		215 (24.2%)	41 (18.1%)		204 (23.8%)	52 (20.2%)	
High school	136 (12.7%)	6 (15.0%)		113 (12.7%)	29 (12.8%)		108 (12.6%)	34 (13.2%)	
College or above	80 (7.4%)	5 (12.5%)		71 (8.0%)	14 (6.2%)		67 (7.8%)	18 (7.0%)	
**Life style**									
Ever smoking (n, %)	289 (26.9%)	6 (15.0%)	0.1	264 (29.7%)	31 (13.7%)	<0.001‡	258 (30.1%)	37 (14.3%)	<0.001‡
Ever drinking (n, %)	128 (11.9%)	2 (5.0%)	0.2	114 (12.8%)	16 (7.1%)	<0.001‡	112 (13.1%)	18 (7.0%)	<0.001‡
Physical activity (MET-h/week)	84.4 (54.4, 148.4)	84.0 (28.0, 140.0)	0.1	84.0 (46.2, 140.0)	102.0 (56.0, 163.8)	0.005†	84.0 (46.2, 141.8)	101.4 (56.0, 158.2)	0.027*
**Body composition indices**									
BMI (kg/m2)	27.4 ± 3.1	27.9 ± 2.6	0.4	27.3 ± 2.9	28.1 ± 3.4	<0.001‡	27.3 ± 3.0	28.0 ± 3.3	<0.001‡
Waist circumference (cm)	93.6 ± 7.1	93.7 ± 5.5	1.0	93.4 ± 6.9	94.6 ± 7.6	0.023*	93.4 ± 7.0	94.3 ± 7.3	0.1
Waist hip ratio	0.937 ± 0.050	0.938 ± 0.049	0.8	0.936 ± 0.050	0.939 ± 0.047	0.5	0.936 ± 0.050	0.938 ± 0.047	0.6
Body fat mass (kg)	23.8 ± 5.3	25.8 ± 6.1	0.021*	23.5 ± 5.3	25.2 ± 5.4	<0.001‡	23.5 ± 5.2	25.2 ± 5.4	<0.001‡
Body fat rate (%)	34.7 ± 6.7	36.5 ± 6.9	0.1	34.1 ± 6.7	37.1 ± 6.4	<0.001‡	34.0 ± 6.7	36.9 ± 6.5	<0.001‡
Body fat free weight (kg)	45.9 ± 8.5	44.8 ± 6.8	0.4	46.4 ± 8.6	43.9 ± 7.5	<0.001‡	46.4 ± 8.6	44.0 ± 7.5	<0.001‡
Muscle mass (kg)	43.4 ± 8.2	42.2 ± 6.6	0.4	43.8 ± 8.2	41.4 ± 7.2	<0.001‡	43.8 ± 8.3	41.5 ± 7.2	<0.001‡
**Clinical characteristics**									
Systolic blood pressure (mmHg)	133.7 ± 17.7	140.5 ± 20.1	0.019*	131.5 ± 16.4	143.6 ± 20.1	<0.001‡	131.2 ± 16.2	143.2 ± 19.9	<0.001‡
Diastolic blood pressure (mmHg)	79.8 ± 10.8	81.2 ± 12.8	0.4	78.8 ± 10.4	83.9 ± 11.5	<0.001‡	78.8 ± 10.4	83.5 ± 11.6	<0.001‡
Serum albumin (g/L)	49.1 ± 2.4	48.4 ± 3.0	0.1	49.0 ± 2.4	49.2 ± 2.4	0.4	49.0 ± 2.4	49.1 ± 2.5	0.6
Serum glubulin (g/L)	25.9 ± 3.4	27.4 ± 3.1	0.009†	25.8 ± 3.4	26.9 ± 3.4	<0.001‡	25.7 ± 3.4	26.9 ± 3.3	<0.001‡
Triglyceride (mmol/L)	1.88 ± 1.29	1.83 ± 0.97	0.8	1.82 ± 1.25	2.10 ± 1.38	0.003†	1.81 ± 1.26	2.06 ± 1.34	0.009†
Total cholesterol (mmol/L)	5.87 ± 1.09	5.88 ± 1.13	0.9	5.81 ± 1.07	6.09 ± 1.14	<0.001‡	5.81 ± 1.06	6.06 ± 1.14	<0.001‡
HDL-cholesterol (mmol/L)	1.37 ± 0.30	1.35 ± 0.26	0.7	1.37 ± 0.30	1.36 ± 0.28	0.6	1.37 ± 0.30	1.36 ± 0.28	0.6
LDL-cholesterol (mmol/L)	3.65 ± 1.02	3.76 ± 0.93	0.5	3.62 ± 1.00	3.78 ± 1.04	0.038*	3.62 ± 1.00	3.78 ± 1.02	0.024*
Fasting glucose (mmol/L)	6.17 ± 1.77	5.84 ± 0.87	0.2	6.05 ± 1.51	6.60 ± 2.44	<0.001‡	6.06 ± 1.53	6.49 ± 2.31	<0.001‡
Glucose 120 min OGTT (mmol/L)	9.06 ± 4.13	8.85 ± 3.32	0.8	8.68 ± 3.80	10.52 ± 4.86	<0.001‡	8.69 ± 3.84	10.26 ± 4.68	<0.001‡
Fasting insulin (mIU/L)	12.7 ± 6.8	13.9 ± 6.1	0.3	12.3 ± 6.2	14.6 ± 8.3	<0.001‡	12.2 ± 6.2	14.5 ± 8.1	<0.001‡
HOMA-IR (*10-6 mol*IU*L-2)	3.54 ± 2.37	3.62 ± 1.72	0.8	3.36 ± 2.06	4.28 ± 3.14	<0.001‡	3.34 ± 2.07	4.21 ± 3.01	<0.001‡
HbA1c	6.21 ± 1.05	6.21 ± 0.65	1.0	6.15 ± 0.94	6.45 ± 1.34	<0.001‡	6.15 ± 0.95	6.41 ± 1.27	<0.001‡
Blood uric acid (mol/L)	360.2 ± 89.6	424.1 ± 119.9	<0.001‡	363.7 ± 90.1	357.5 ± 97.3	0.4	361.4 ± 87.9	366.0 ± 102.9	0.5
Hyperuricemia, (n (%))	404 (37.6%)	26 (65.0%)	<0.001‡	331 (37.2%)	99 (43.8%)	0.1	310 (36.2%)	120 (46.5%)	0.003†
Irisin (ng/mL)	7.29 (2.72, 14.82)	6.46 (2.84, 11.05)	0.5	7.18 (2.76, 15.15)	7.74 (2.58, 13.30)	0.8	7.18 (2.74, 15.23)	7.53 (2.74, 13.13)	0.6
Irisin-log transformed (ng/mL)§	1.30 ± 1.24	1.28 ± 1.04	1.0	1.31 ± 1.22	1.23 ± 1.29	0.6	1.31 ± 1.23	1.24 ± 1.25	0.6

*p < 0.05, †p < 0.01, ‡p < 0.001.

§Irisin was log-transformed to obtain better approximation of normal distribution.

All percentages are column percentage; except for percentages, all values are mean ± s.d. or median(25th, 75th) for non-normal distribution data.

*Abbreviations*: *A/G* ratio, albumin/globulin ratio, *BMI* body mass index, *CKD* chornic kidney disease, *eGFR* estimated glomerular filtration rate, *HDL* high-density lipoprotein cholesterol, *HOMA-IR* homeostasis model assessment insulin resistance index, *LDL* low-density lipoprotein cholesterol, *MET-h/week* metabolic equivalent hours per week.

### Demographic and clinical characteristics stratified by quartiles of serum irisin

Table 2 shows that subjects were divided into four groups according to quartiles of serum irisin levels (median (interquartile range): 0.13(0.02 – 1.56), 4.73(3.83 – 5.97), 10.38(8.67 – 12.17) and 23.05(17.73 – 31.28) for quartile 1–4, respectively). With increasing levels of serum irisin, subjects had significantly lower levels of waist hip ratio and muscle mass but higher levels of serum globulin and LDL and more likely to be females. There were no statistically significant difference in the levels of systolic BP, diastolic BP, BMI, waist circumference, body fat percentage, TG, TC, HDL, FPG, fasting insulin, HOMA-IR and HbA1c among these four groups. Subjects within the quartile 4 group of serum irisin had significantly the lowest prevalence rate of CKD (22.9%, 22.2%, 28.7% and 18.7% for quartile 1–4 groups, respectively, p = 0.046); and they also showed the lowest prevalence rate of albuminuira, although the difference was not statistically significant (20.4%, 18.6%, 25.1% and 16.9% for quartile 1–4 groups, respectively, p = 0.093).

**Table 2:** **Demographic, lifestyle and clinical characteristics of subjects by tertiles of irisin**

**Variables**	**Quartile 1**	**Quartile 2**	**Quartile 3**	**Quartile 4**	**Total**	**P value**
Irisin (ng/mL)	0.13 (0.02, 1.56)	4.73 (3.83, 5.97)	10.38 (8.67, 12.17)	23.05 (17.73, 31.28)	7.26 (2.75, 14.72)	<0.001‡
**Demographics**						
N (%)	279 (25.0%)	279 (25.0%)	279 (25.0%)	278 (25.0%)	1115 (100.0%)	
Sex						
Male (n, %)	94 (33.7%)	100 (35.8%)	85 (30.5%)	70 (25.2%)	349 (31.3%)	0.039*
Female (n, %)	185 (66.3%)	179 (64.2%)	194 (69.5%)	208 (74.8%)	766 (68.7%)	
Age (years)	53.2 ± 7.4	52.6 ± 7.4	53.4 ± 7.1	53.5 ± 7.0	53.2 ± 7.2	0.7
Education categories, (n, %)						0.4
Illiteracy	66 (23.7%)	70 (25.1%)	91 (32.6%)	75 (27.0%)	302 (27.1%)	
Elementary school	87 (31.2%)	77 (27.6%)	77 (27.6%)	89 (32.0%)	330 (29.6%)	
Middle school	67 (24.0%)	77 (27.6%)	52 (18.6%)	60 (21.6%)	256 (23.0%)	
High school	35 (12.5%)	33 (11.8%)	38 (13.6%)	36 (13.0%)	142 (12.7%)	
College or above	24 (8.6%)	22 (7.9%)	21 (7.5%)	18 (6.4%)	85 (7.6%)	
**Life style**						
Ever smoking (n, %)	82 (29.4%)	81 (29.0%)	73 (26.2%)	59 (21.2%)	295 (26.5%)	0.1
Ever drinking (n, %)	37 (13.3%)	27 (9.7%)	36 (12.9%)	30 (10.8%)	130 (11.7%)	0.4
Physical activity (MET-h/week)	84.0 (46.2, 158.2)	84.0 (46.2, 140.0)	87.3 (46.2, 152.4)	100.3 (56.0, 154.0)	84.0 (51.6, 148.4)	0.4
**Body composition indices**						
BMI (kg/m2)	27.5 ± 3.1	27.3 ± 3.1	27.7 ± 3.3	27.2 ± 2.8	27.5 ± 3.1	0.2
Waist circumference (cm)	94.1 ± 7.3	93.8 ± 7.2	93.4 ± 7.1	93.1 ± 6.6	93.6 ± 7.1	0.4
Waist hip ratio	0.940 ± 0.049	0.943 ± 0.050	0.933 ± 0.050	0.930 ± 0.049	0.937 ± 0.049	0.006†
Body fat mass (kg)	24.0 ± 5.4	23.3 ± 5.3	24.1 ± 5.6	24.0 ± 5.1	23.9 ± 5.3	0.2
Body fat rate (%)	34.6 ± 6.6	34.0 ± 6.9	35.1 ± 7.0	35.3 ± 6.3	34.7 ± 6.7	0.1
Body fat free weight (kg)	46.7 ± 9.2	46.2 ± 8.4	45.8 ± 8.5	44.8 ± 7.5	45.9 ± 8.4	0.1
Muscle mass (kg)	44.1 ± 8.8	43.7 ± 8.1	43.2 ± 8.1	42.2 ± 7.2	43.3 ± 8.1	0.048*
**Clinical characteristics**						
Systolic blood pressure (mmHg)	133.9 ± 17.5	133.1 ± 17.6	135.7 ± 20.1	133.1 ± 16.0	134.0 ± 17.9	0.3
Diastolic blood pressure (mmHg)	80.1 ± 10.7	80.0 ± 10.8	80.4 ± 12.1	79.0 ± 9.6	79.9 ± 10.8	0.4
Serum albumin (g/L)	49.1 ± 2.4	48.9 ± 2.4	49.0 ± 2.3	49.2 ± 2.5	49.0 ± 2.5	0.8
Serum glubulin (g/L)	25.0 ± 3.2	25.7 ± 3.4	26.5 ± 3.7	26.8 ± 3.4	26.0 ± 3.4	<0.001‡
Triglyceride (mmol/L)	1.94 ± 1.44	1.98 ± 1.34	1.81 ± 1.16	1.77 ± 1.15	1.87 ± 1.28	0.2
Total cholesterol (mmol/L)	5.83 ± 1.10	5.77 ± 1.00	5.91 ± 1.11	5.95 ± 1.13	5.87 ± 1.09	0.2
HDL-cholesterol (mmol/L)	1.36 ± 0.30	1.35 ± 0.31	1.36 ± 0.28	1.39 ± 0.30	1.37 ± 0.30	0.6
LDL-cholesterol (mmol/L)	3.59 ± 1.02	3.54 ± 0.96	3.73 ± 1.03	3.76 ± 1.03	3.65 ± 1.01	0.022*
Fasting glucose (mmol/L)	6.22 ± 1.95	6.09 ± 1.69	6.23 ± 1.95	6.09 ± 1.35	6.16 ± 1.75	0.6
Glucose 120 min OGTT (mmol/L)	9.11 ± 4.17	8.93 ± 4.09	9.16 ± 4.24	9.01 ± 3.90	9.05 ± 4.10	0.9
Fasting insulin (mIU/L)	13.2 ± 7.2	12.6 ± 6.8	12.5 ± 6.4	12.7 ± 6.6	12.7 ± 6.8	0.6
HOMA-IR (*10-6 mol*IU*L-2)	3.68 ± 2.37	3.49 ± 2.59	3.51 ± 2.22	3.50 ± 2.21	3.54 ± 2.35	0.7
HbA1c	6.27 ± 1.25	6.17 ± 0.97	6.23 ± 1.06	6.16 ± 0.85	6.21 ± 1.04	0.6
Hyperuricemia, (n (%))	120 (43.0%)	103 (36.9%)	104 (37.3%)	103 (37.1%)	430 (38.6%)	0.4
eGFR <60 mL/min/1.73 m2, (n (%))	9 (3.2%)	13 (4.7%)	11 (3.9%)	7 (2.5%)	40 (3.6%)	0.6
Albumin creatinine ratio > =30 mg/g, (n (%))	57 (20.4%)	52 (18.6%)	70 (25.1%)	47 (16.9%)	226 (20.3%)	0.1
CKD, (n (%))	64 (22.9%)	62 (22.2%)	80 (28.7%)	52 (18.7%)	258 (23.1%)	0.046*

*p < 0.05, †p < 0.01, ‡p < 0.001.

All percentages are column percentage; except for percentages, all values are mean ± s.d. or median(25th, 75th) for non-normal distribution data.

*Abbreviations*: *ACR* albumin creatinine ratio, *BMI* body mass index, *CKD* chornic kidney disease, *eGFR* estimated glomerular filtration rate, *HDL* high-density lipoprotein cholesterol, *HOMA-IR* homeostasis model assessment insulin resistance index, *LDL* low-density lipoprotein cholesterol, *MET-h/week* metabolic equivalent hours per week.

### Associations of serum irisin and body composition measurements with reduced renal function, albuminuira and CKD

Table 3 shows the adjusted odds ratios (ORs) with associated 95% confidence interval (CI) of serum irisin and body composition measurements for reduced renal function, albuminuria and CKD. In model 1 with adjustment for sex and age, increasing levels of body composition measurements, such as BMI, waist circumference, body fat mass and body fat percentage, were all significantly associated with increased risks of albuminuria and CKD. Body fat mass and body fat percentage were also significantly associated with increased risk of reduced renal function. Waist hip ratio, body fat free weight and muscle mass were not significantly associated with reduced renal function, albuminuria and CKD (data not shown). Serum irisin concentration (both the log-transformed value and the categorical values) was not significantly associated with reduced renal function, albuminuria or CKD. In model 2 (additional adjustments for educational level, smoking and drinking habits and physical activity), the results for body composition measurements were quite similar to those in model 1. However, subjects with the highest serum irisin (quartile 4 group) showed significantly decreased risk of CKD compared with those with the lowest (quartile 1 group), with the adjusted OR (95% CI) of 0.620 (0.393-0.980, p = 0.041).

**Table 3:** **Adjusted odds ratios (ORs) with associated 95% confidence interval (CI) for reduced renal function, albuminuira and CKD**

	**Reduced renal function**			**Albuminuira**			**CKD**		
**Variables**	**OR**	**95% CI**	**P value**	**OR**	**95% CI**	**P value**	**OR**	**95% CI**	**P value**
**Model 1**									
BMI (kg/m2)	1.073	0.971-1.186	0.2	1.09	1.047-1.148	<0.001*	1.098	1.050-1.149	<0.001*
Waist circumference (cm)	0.995	0.947-1.045	0.8	1.039	1.018-1.061	<0.001*	1.031	1.011-1.052	0.003*
Body fat mass (kg)	1.084	1.023-1.150	0.007*	1.049	1.018-1.080	0.002*	1.055	1.026-1.086	<0.001*
Body fat rate (%)	1.083	1.004-1.167	0.038*	1.082	1.043-1.121	<0.001*	1.085	1.048-1.124	<0.001*
Serum irisin†	0.987	0.721-1.353	0.9	0.948	0.820-1.096	0.5	0.949	0.826-1.091	0.5
Serum irisin									
(Quartile 2 vs. Quartile 1)	1.616	0.669-3.906	0.3	0.926	0.605-1.416	0.7	1.007	0.671-1.511	1.0
(Quartile 3 vs. Quartile 1)	1.228	0.493-3.057	0.7	1.287	0.859-1.926	0.2	1.340	0.908-1.979	0.1
(Quartile 4 vs. Quartile 1)	0.766	0.278-2.110	0.6	0.740	0.479-1.141	0.2	0.721	0.474-1.096	0.1
**Model 2**									
BMI (kg/m2)	1.074	0.958-1.205	0.2	1.100	1.046-1.156	<0.001*	1.102	1.049-1.158	<0.001*
Waist circumference (cm)	1.011	0.959-1.066	0.7	1.038	1.015-1.061	0.001*	1.034	1.011-1.057	0.003*
Body fat mass (kg)	1.093	1.020-1.172	0.012*	1.053	1.019-1.087	0.002*	1.059	1.026-1.093	<0.001*
Body fat rate (%)	1.088	1.000-1.183	0.1	1.083	1.042-1.125	<0.001*	1.087	1.047-1.129	<0.001*
Serum irisin †	0.992	0.697-1.412	1.0	0.914	0.784-1.066	0.3	0.917	0.859-1.379	0.3
Serum irisin									
(Quartile 2 vs. Quartile 1)	1.738	0.640-4.722	0.3	0.760	0.477-1.209	0.2	0.848	0.542-1.326	0.5
(Quartile 3 vs. Quartile 1)	1.334	0.471-3.780	0.6	1.106	0.711-1.719	0.7	1.191	0.775-1.831	0.4
(Quartile 4 vs. Quartile 1)	0.607	0.183-2.008	0.4	0.641	0.401-1.024	0.1	0.620	.392-0.980	0.041*
**Model 3**									
BMI (kg/m2)	1.031	0.910-1.167	0.6	1.053	0.996-1.113	0.1	1.052	0.996-1.110	0.1
Waist circumference (cm)	0.997	0.945-1.053	0.9	1.023	0.998-1.048	0.1	1.017	0.993-1.041	0.2
Body fat mass (kg)	1.076	0.999-1.159	0.1	1.034	0.998-1.071	0.1	1.039	1.004-1.076	0.029*
Body fat rate (%)	1.062	0.969-1.164	0.2	1.046	1.002-1.092	0.039*	1.049	1.006-1.093	0.025*
Serum irisin†	0.946	0.660-1.357	0.8	0.910	0.771-1.073	0.3	0.906	0.772-1.064	0.2
Serum irisin									
(Quartile 2 vs. Quartile 1)	1.609	0.579-4.472	0.4	0.786	0.485-1.272	0.3	0.873	0.549-1.388	0.6
(Quartile 3 vs. Quartile 1)	1.185	0.407-3.448	0.8	1.050	0.658-1.673	0.8	1.123	0.713-1.769	0.6
(Quartile 4 vs. Quartile 1)	0.507	0.147-1.746	0.3	0.611	0.373-1.000	0.050*	0.572	0.353-0.927	0.023*

*p < 0.05.

†OR and 95% CI was impressed as per SD increase of log transformed serum irisin.

*Abbreviations*: *BMI* body mass index, *CKD* chornic kidney disease.

Model 1 was adjusted for sex and age;

Model 2 was further adjusted for educational level, ever smoking, ever drinking and physical activity.

Model 3 was further adjusted for SBP, DBP, serum glubulin, hyperuricemia,TG,LDL, fasting glucose.

The last full model (model 3) was further adjusted for SBP, DBP, serum globulin, hyperuricemia, TG, LDL and FPG. As for body composition measurements, increasing body fat percentage was significantly associated with increased risks of both albuminuira and CKD, and increasing body fat mass was also significantly associated with increased risk of CKD. Other body composition measurements, such as BMI and waist circumference, were not significantly associated with reduced renal function, albuminuria or CKD. Compared with those with the lowest serum irisin (quartile 1 group), subjects with the highest (quartile 4 group) showed significantly decreased risk of CKD and marginally decreased risk of albuminuria, with the adjusted ORs (95% CI) of 0.572 (0.353-0.927, p = 0.023) and 0.611 (0.373-1.000, p = 0.050), respectively. We also used log transformation of eGFR and ACR for linear regression analysis, but the associations of serum irisin with eGFR and ACR were not statistically significant (data not shown).

No statistically significant interaction effect between serum irisin and body composition measurements on CKD was found. Furthermore, stratified analysis by diabetes mellitus showed similar results of the association between serum irisin and CKD as those in Table 3 (data not shown).

## Discussion

Obesity has become a worldwide epidemic, and the prevalence of obesity-related health conditions, such as insulin resistance, metabolic syndrome, type 2 diabetes, hypertension, cardiovascular disease, cancer and chronic kidney disease, increased significantly [9,10]. Physical exercise, as a lifestyle intervention approach, has long been recognized for its effects on these obesity-related comorbidities, although the underlying mechanisms remain to be fully understood [32]. Irisin was identified in 2012 by Boström as a putative exercise induced myokine, which drives brown-fat-like conversion of white adipose tissues, has broad implications for metabolism and energy homeostasis [13,15]. Circulating irisin has been found to be reduced in nonalcoholic fatty liver disease (NAFLD) [27] and type 2 diabetes patients compared with their controls [15-17]. Because NAFLD, type 2 diabetes and CKD share the same pathology of obesity and insulin resistance [33], it is therefore hypothesized that serum irisin level was negatively associated with risk of CKD.

Wen and co-workers first found that plasma irisin levels were significantly decreased in 38 CKD patients compared to 19 age- and sex-matched normal controls, but they failed to find independent association of irisin with CKD [18]. Liu found that circulating irisin was significantly decreased in 179 T2DM patients with renal insufficiency (eGFR < 60 ml/min/1.73 m^2^) compared to 186 T2DM patients with normal renal function (eGFR ≥ 60 ml/min/1.73 m^2^); and the reduction in irisin was most pronounced in stage 5 CKD patients [19]. In the multivariate linear regression model, Liu found that circulating irisin was independently associated with GFR but failed to observe association of circulating irisin with early markers of kidney injury such as microalbuminuria [19]. In 532 patients with stages 1–5 of CKD, Ebert found that irisin serum concentrations decreased with increasing CKD stage and are independently and positively predicted by renal function in robust multivariate analysis [20]. However, several major limitations diminished the value of these studies, such as the relatively small sample sizes and including only CKD patients or T2DM patients. Larger studies with different populations are awaited to validate the associations of serum irisin concentration with CKD.

In the present study, we found that, compared to those with the lowest serum irisin (quartile 1 group), subjects with the highest (quartile 4 group) had significantly the lowest prevalence of CKD. In multivariable logistic regression model with adjustment for potential confounders, compared with those within quartile 1 group of serum irisin, subjects within quartile 4 group showed significantly decreased risk of CKD and marginally decreased risk of albuminuria, although subjects in quartile 2–3 groups failed to show similar trend. Therefore, further studies on the association between serum irisin and CKD with larger sample size from different populations, especially from the prospective cohort studies are warranted.

Mechanisms underlying the association between serum irisin and decreased risk of CKD are not fully understood. Firstly, some suggest that CKD is characterized by progressive muscle wasting and reduced muscle mass may partially account for reduced irisin expression and secretion. However, in our previous paper, we did not find a positive relationship between serum irisin and body muscle mass [26]. Furthermore, in the multivariable logistic regression model in the present study, muscle mass was adjusted for as a potential confounder. So it seems our results do not support this hypothesis. Secondly, insulin resistance and hyperglycemia play important roles in patients with CKD [33]. In our previous paper, we found that fasting insulin and HbA1c were negatively associated with serum irisin level in stepwise multivariable linear regression analysis [26]. Therefore, our results seem to support the hypothesis that irisin improves insulin resistance and glucose homeostasis and then reduces the risk of CKD. Our results may have clinical implication of the therapeutic potential of irisin for CKD if further studies, especially from prospective cohort studies and intervention studies, clearly demonstrate the protective effect of irisin on CKD.

Numerous previous studies have found that obesity is independently associated with increased risk of CKD, and body mass index (BMI) has been widely used as the index of obesity in these studies [1-3,5-7]. However, there are still many studies which have shown that BMI and other measures of adiposity, such as waist circumference, body fat percentage, have different associations with various health conditions [21-23]. Furthermore, recent studies show that irisin is not only a myokine but also an adipokine, both muscle and adipose tissue contribute to circulating irisin [24,25]. These two points prompt us to test the independent association of different body composition measurements with CKD. And we found that only body fat percentage, but not BMI or waist circumference, was independently associated with CKD after adjusting for potential confounders. Our results suggest that adiposity per se, rather than body weight or body shape, is independently associated with increased risk factor of CKD. Therefore, more studies are warranted to test the independent association of different measures of body composition with CKD in different populations.

China, a developing country with a relatively high economic development speed within the past three decades, is facing the increasing prevalence of obesity and associated health conditions, such as cardiovascular disease, diabetes and CKD. Although the prevalence of CKD in China has been reported to be around 10% [5-7], little evidence is available on the prevalence of CKD in obese Chinese adults. The present study found that, for the obese healthy Chinese adults, the prevalence rates (95%CI) of CKD were 26.6% (23.2%-29.4%) in females and 15.5% (11.8%-19.2%) in males, which were much higher than those of the general adults in China. We also found that the prevalence of albuminuria (23.8% in females and 12.6% in males) was much higher than those of general Chinese adults (6%-9%) [5-7]. Therefore, for the obese adults in China, even they may look generally healthy, screening for CKD, especially albuminuria, should attract attention not only for heath workers but also for obese subjects themselves.

Several limitations of the present study need to be recognized. First, the present subjects were not randomly sampled from their living communities and were generally healthy without any previously diagnosed common chronic diseases. Those who had serious chronic diseases were underrepresented in the present study; so there were some kinds of “healthy volunteer bias” in the present study, and we might under-estimate the prevalence of CKD in the general obese adults. Therefore, the present sample was not as good as that which was randomly sampled from the general community-living subjects to estimate the prevalence of CKD. Second, we are not certain about the temporal sequence between serum irisin and CKD because of the cross-sectional study design. Therefore, our results should be confirmed in future prospective cohort studies and intervention studies. Third, all subjects recruited into the present study are central obese adults, which may hamper us to extrapolate our findings to the non-obese adults. Last but not least, we did not measure other cytokines, such as adiponectin, IL-6 and TNF-α, which may be related to serum irisin. On the other hand, our study has a lot of strengths. Firstly, our study has a relatively large sample size of obese adults. And we have also adjusted for much more potential confounders than previous studies. For example, few previous studies adjusted for physical activity, body fat and muscle mass, which were important confounding factors of serum irisin.

## Conclusions

Our study found that subjects with the highest serum irisin (quartile 4 group) had significantly decreased risk of CKD and marginally decreased risk of albuminuria. Future works about the association of serum irisin with CKD, especially from prospective cohort studies with larger sample size, are warranted. In addition, we also found only body fat percentage, neither BMI nor waist circumference, was independently associated with increased risk of CKD, which suggests that it is adiposity per se, rather than body weight or body shape, is independently associated with increased risk of CKD. Therefore, decreasing body fat percentage should be emphasized for the obese adults. Third, the prevalence of CKD in obese adults was much higher than that of the general adults in China; of which the prevalence of albuminuria was about two or three times of those general adults. Therefore, screening for CKD, especially albuminuria, in the obese adults, should attract attention not only for heath workers but also for obese subjects themselves.

## Abbreviations

BMI: Body mass index
CKD: Chronic kidney disease
HDL: High-density lipoprotein cholesterol
HOMA-IR: Homeostasis model assessment insulin resistance index
LDL: Low-density lipoprotein cholesterol
MET-h/week: Metabolic equivalent hours per week
